# The Importance of the Social Sciences in Reducing Tail Biting Prevalence in Pigs

**DOI:** 10.3390/ani9090591

**Published:** 2019-08-21

**Authors:** Grace A. Carroll, Jenny M. Groarke

**Affiliations:** 1 Animal Behaviour Centre, School of Psychology, Queen’s University Belfast, David Keir Building, 18–30 Malone Road, Belfast BT9 5BN, Northern Ireland, UK; 2 Centre for Improving Health Related Quality of Life (CIHRQoL), School of Psychology, Queen’s University Belfast, David Keir Building, 18–30 Malone Road, Belfast BT9 5BN, Northern Ireland, UK

**Keywords:** tail biting, pig welfare, health psychology, behaviour change, intervention

## Abstract

**Simple Summary:**

Tail lesions are a major welfare concern within pig farming. Tail lesions result from biting and chewing of the tail of one pig by another and can indicate boredom and frustration within the herd. While extensive research has been carried out to understand and eliminate tail biting in pigs, findings from scientific studies have often not been applied in practice. This may be due, in part, to a failure to consider the role of farmer behaviour in improving animal welfare. If farmer behaviour does not change, it is unlikely that tail lesion prevalence will change from current levels. In this paper, the shortcomings of traditional behaviour change theories were discussed and a guide for designing human behaviour change interventions for pig farmers was provided. It is essential that collaborations between social scientists and animal welfare scientists occur if research findings are to be put into practice on farms.

**Abstract:**

Tail biting in pigs has been recognised as a welfare problem for several decades, being referred to in scientific literature as far back as the 1940s. Today, animal welfare scientists have a solid understanding of the aetiology of tail biting. Despite this, there has been a major failure in applying research findings on commercial farms. Consequently, tail biting remains a significant problem in modern intensive pig farming. Of all farming industry stakeholders, farmers have the greatest influence over the welfare of their animals. Despite this, little animal welfare research has focused on changing farmer behaviour. Understanding the reasons why farmers act or fail to act to improve animal welfare is key if research findings are to be translated into practical on-farm change. Adopting the principles of behavioural science, this review discussed theory-based methods of identifying barriers to effective tail biting management. A guide was provided for designing behaviour change interventions for farmers using The Behaviour Change Wheel, a systematic framework that links the source of behaviour to suitable interventions. It was concluded that the social sciences are of great importance to ensuring that theory is put into practice.

## 1. Introduction

Tail lesions are a major welfare concern, resulting from biting and chewing of the tail of one pig by another. Tail lesions range in severity, ending in complete loss of the tail in the most serious cases [1,2]. Tail biting in pigs has been recognised as a welfare problem for several decades, being referred to in the scientific literature as far back at the 1940s [3]. Studies from the 1960s and 1970s discuss procedures for dealing with tail biting, such as removing the biter or the bitten pig, and the role of plentiful bedding in reducing the problem [4,5]. In 1982, van den Berg stated that while there was a good understanding of the causes and prevention of tail biting, it remained a common vice in pigs [6]. Almost 20 years later, Schroder-Petersen and Simonsen (2001) [3] reported that the problem with tail biting was at least as severe as it was 50 years ago. Fast-forward another 20 years and tail biting remains a significant issue for both animal welfare and farm productivity; recent research has highlighted the link between tail lesions and productivity-related parameters, such as increased partial carcass condemnations, decreased cold carcass weights, reduced lean meat content and increased carcass abscessation [7,8,9,10,11]. Furthermore, tail-bitten pigs grow more slowly, often requiring them to be kept on farms for longer periods until the necessary live weight has been reached. This increases feed costs and reduces space available for incoming animals. Therefore, reducing tail lesions in pig herds will have positive benefits in economic terms. However, despite the extensive research on the subject, there appears to be a lack of understanding among pig farmers of the economic cost of tail biting [12].

In the field of Farm Animal Welfare Science, environmental enrichment strategies, alternative housing systems and precision livestock farming methods have been developed to address welfare concerns [13,14]. However, there has been a failure in applying research findings and technological advances on commercial farms, and this is being increasingly recognised by the scientific community [15,16]. While scientists are interested in addressing broad issues, farmers are typically more interested in solving problems that are directly relevant to them [17]. Furthermore, involving farmers in the research process can be difficult when this interferes with the farmer’s daily work routine [18]. As a result, research scientists and product developers often fail to consult with farmers in the early stages of development [16,19]. Consequently, there may not be demand for a specific product within the farming community and the technology involved may be too complex. Furthermore, farmers are often unwilling to invest in new methods and technologies when profit margins are small and the financial payoff is not immediate. Therefore, a significant amount of money has been spent on concepts developed by animal welfare scientists that have never been adopted in practice [20]. It is acknowledged that tail biting outbreaks are difficult to predict and because of this, farmers can often only respond once the outbreak has begun [21,22]. Furthermore, although less common, tail biting can be seen in outdoor systems as well as in intensive indoor systems (e.g., [23]). It is therefore likely that there will always be some level of tail biting present within commercial pig herds. Nonetheless, of all farming industry stakeholders, farmers have the greatest influence over the welfare of their animals [15] and understanding the reasons why farmers act or fail to act to improve animal welfare is key if research findings are to be translated into practical on-farm change.

Moreover, in 2017, the EU Commission launched an action plan to facilitate an end to routine tail docking. Tail docking is carried out to reduce the risk of tail biting and a recent report by Briyne et al. (2018) [24] suggests that, with some exceptions, 81%–100% of EU pigs are being docked routinely. Indeed, over 99% of pigs in the Republic of Ireland (ROI) and Northern Ireland (NI) are tail docked [25]. EU member states have been required to develop tail docking action plans and increased attention on this issue is leading to greater enforcement of the tail docking ban [24]. July 2019 marks the end of the action plan timeline, with infringement procedures for non-compliance by Member States being a potential outcome [26]. As tail docking is one of the most common ways of reducing tail lesion prevalence, there is fear that enforcement of the ban will lead to an upsurge in tail lesion prevalence across Member States. For example, Lahrmann et al. (2017) [27] examined tail lesion prevalence in docked versus undocked pigs in a Danish herd with a typically low prevalence of tail biting. Lehrmann et al. (2017) [27] found that 23% of undocked pigs were observed to have a lesion between weaning and slaughter, compared to 0% of docked pigs. Given this, it is particularly pertinent that farmers act to address the issue of tail biting on their farms. However, Carroll et al. (2016) [7] found that 30.8% of pigs across two Irish abattoirs had tail lesions, despite being tail docked. Therefore, while greater enforcement of the tail docking ban will increase the risk for tail biting, tail biting remains a problem in systems both with and without routine tail docking, as well as in both outdoor and intensive indoor systems.

## 2. Behaviour Change Research in Animal Welfare and Agricultural Research to Date

Behaviour change frameworks were originally developed within the field of health psychology and have been applied to human health-related behaviour such as smoking cessation, medication adherence and weight loss [28]. A number of theories such as the Protection Motivation Theory [29], the Theory of Reasoned Action [30], Goal-Setting Theory [31], the Health Belief Model [32] and the often-cited Theory of Planned Behaviour [33] have informed behavioural intervention design [34]. One review found that interventions can increase self-efficacy (i.e., belief in one’s own ability to achieve goals) and that this increase was related to subsequent health behaviour change [35]. Importantly, a greater use of theory has been associated with increased effect sizes in behaviour change interventions and interventions based on the Theory of Planned Behaviour had particularly strong effects on health behaviour change [36].

The Theory of Planned Behaviour has been the dominant theory guiding research on health-related behaviour for the last 30 years. Indeed, the Theory of Planned Behaviour has been cited in hundreds of papers related to the discipline of animal welfare science. The Theory of Planned Behaviour is based on the idea that intention to perform a behaviour is determined by (i) attitude, (ii) subjective norm (e.g., social pressure) and (iii) perceived behavioural control. Intentions, in turn, determine behaviour [33]. Therefore, according to this theory, attitudes towards animal welfare should predict farmer behaviour and consequently, farm animal welfare status. This idea has given rise to a number of studies that focused on assessing farmer attitudes as a way of understanding and potentially improving animal welfare standards on-farm. For example, Kauppinen et al. (2010) [37] assessed pig and dairy farmers’ perceptions of animal welfare via a questionnaire that was designed using the Theory of Planned Behaviour. Kauppinen et al. (2010) [37] found that positive attitudes were associated with the intention to treat animals in a humane manner. Similarly, Borges et al. (2019) [38] examined pig farmers decisions surrounding the adoption of environmental enrichment on-farm. Using the Theory of Planned Behaviour as a theoretical framework, Borges et al. (2019) [38] found that perceived behavioural control, that is, the perception of having the ability/resources to add enrichment, was the chief determinant of pig farmers’ intentions to provide enrichment to their animals. The perceived effects on animal stress and farm productivity were also determinants of intention to provide environmental enrichment. However, while studies that focus on farmer intentions may be useful, limitations of the Theory of Planned Behaviour have also been highlighted [39]. In particular, an intention-behaviour gap exists in that what people intend to do does not determine what they actually do [40]. This gap has been widely acknowledged within consumer psychology, where decision aspects such as convenience, habit and price supersede the intention to purchase ethically sourced food [41,42]. Similarly, farmers may intend to improve animal welfare but other decision criteria, such as time considerations and workload, take precedence. Furthermore, when farmers express their attitudes and intentions, a number of biases may influence how they respond. For example, farmers’ responses during interviews or via questionnaires may be influenced by a desire to be viewed in a positive light by the researcher or society in general. Furthermore, too little time may be allocated to considering and responding to a posed question and the farmer may be trying to influence the outcome of the study through their responses [43,44]. The latter may occur when farmers believe that the study results will be used to inform future policy decisions [44].

Lefebvre et al. (2014) [44] examined farmer land investment intentions as a case study of farmer decision making. Lefebvre et al. (2014) [44] found that 74% of farmers acted as intended within 3 years of stating their intention. However, the majority of farmers had stated that their intention was *not* to invest, i.e., to do nothing. In contrast, only 36% of farmers that intended to invest actually did so. Therefore, it is possible that intentions may only be a reliable indicator of future behaviour when the intention is a passive one requiring no further action. Consequently, it is important to determine whether farmers with positive attitudes and intentions towards animal welfare do indeed behave in a manner that promotes animal welfare. Kauppinen et al. (2012) [45] examined associations between piglet productivity parameters, such as piglet mortality, and farmer attitudes towards improving animal welfare. A number of associations were found. For example, a positive attitude towards the provision of a suitable environment was associated with decreased piglet mortality between birth and weaning. However, most effects were only seen in primiparious litters and all the identified associations were weak (r < 0.300). In addition, the association between positive attitude and piglet mortality could have been affected, in part, by timely culling of sick or injured pigs by farmers with a more positive attitude towards animal welfare. In a similar study, Kielland et al. (2010) [46] aimed to determine how farmers’ attitudes and empathy were associated with mastitis, fertility, milk production and skin lesions in dairy cattle. Kielland et al. (2010) [46] found that one group of farmers that were high on empathy and had a positive attitude also had lower herd-level skin lesions compared to other groups. However, this group also had a lower milk yield compared to the other identified groups. In this study, farmer attitude was measured using one Likert-scale question on the ability of animals to experience pain and therefore cannot be interpreted as reflecting attitudes toward animals more generally. Kiliç and Bozkurt (2013) [47] found that Turkish sheep farmers that provided licking blocks, trained their personnel, and reported that they did not have a problem with mastitis had more positive perceptions of animal welfare than farmers that did not report this. Likewise, O’Kane et al. (2017) [48] assessed the association between lameness prevalence in sheep and farmer traits such as personality, emotions, and attitudes and beliefs about footrot. A number of associations were found between lameness prevalence and emotions towards footrot, such as feelings of hopelessness in tackling the issue. Unfortunately, neither study [47,48] used objective measures of animal health and welfare, instead relying on farmers’ self-reporting of mastitis and lameness in their herds. Therefore, the precise link between farmers’ attitudes, behaviour, and animal welfare in these cases remains unclear.

Welfare outcomes need to be measured directly and validated methods of assessing farmer attitudes need to be developed and implemented. In addition, it is important that factors other than attitudes and intentions be considered when aiming to change human behaviour; a major criticism of the Theory of Planned Behaviour [33] is that it fails to consider aspects of human decision making such as moral norms, motivation, culture and social and economic constraints encountered in the real world [34]. For example, pig welfare issues cannot be fully separated from market forces, government regulations, and changing consumer demands [49,50]. Furthermore, the physical characteristics of the farm will determine the level of time and labour needed to make changes to the current system [51]. Therefore, a holistic approach is needed which considers both intrinsic and extrinsic factors affecting farmer behaviour. Moreover, the Theory of Planned Behaviour does not provide an explanation of how cognition changes, making it difficult to utilise in the development of behaviour change interventions [39].

In recent years, behavioural science has moved away from adopting a single theoretical framework, towards widespread adoption of frameworks based on a synthesis of available theories and models, more specifically, the Theoretical Domains Framework [52], the Behaviour Change Wheel [53] and the Behaviour Change Techniques Taxonomy [54]. These frameworks are described in the next section, along with guidance for applying these models to design effective interventions to change farmer behaviour to reduce tail biting in pigs.

## 3. A Guide for Designing Behaviour Change Interventions to Reduce Tail Biting

This section describes the three stages in the development of behaviour change interventions: (i) understand the behaviour (ii) identify intervention options, and (iii) identify intervention content and implementation options. A summary of the main features and uses of the Theoretical Domains Framework (TDF) [52], Behaviour Change Wheel (BCW) [53] and the Behaviour Change Techniques Taxonomy (BCTT) [54] was provided for reference and can be seen in Table 1.

### 3.1. Understand the Behaviour

The first step in developing an intervention involves understanding the behaviour; defining the problem in behavioural terms, selecting the target behaviour, and identifying what specifically needs to be changed. When developing an intervention, the intervention developer must first understand all the potential behaviours that come to bear on a specific problem (e.g., tail biting) and select an appropriate target behaviour for their intervention. In the case of tail biting interventions, the developer could be an animal welfare scientist, agricultural advisor or government body responsible for ensuring compliance with farm animal welfare legislation. The success of behaviour change interventions can be determined on multiple levels. For example, a behaviour change intervention to reduce tail biting could target a specific farmer behaviour, such as the identification and removal of the biter. An intervention might also focus on changing an outcome, such as reducing tail lesion prevalence via encouraging farmers to reduce stocking density or improving air quality. Next, to understand what specifically needs to change, the barriers to and facilitators of the target behaviour should be identified. This is most often carried out via literature reviews, interviews, focus groups and questionnaires [51].

The Theoretical Domains Framework [52] can be applied to support this process. The Theoretical Domains Framework is an amalgamation of 33 different theories of behaviour change developed through expert consensus. Fourteen theoretical domains that influence behaviour were identified, including knowledge, skills, social/professional role and identity, beliefs about capabilities, optimism, beliefs about consequences, reinforcement, intention, goals, memory, attention and decision processes, environmental context and resources, social influences, and emotion and behavioural regulation [55]. These domains can be used to design survey items and interview questions to systematically identify potential barriers and facilitators of a desired behaviour [56,57,58]. For example, the influence of ‘beliefs about consequences’ on farmer behaviour could be examined by asking the farmer to discuss their perception of the economic consequences of tail biting. 

**Figure 1:**
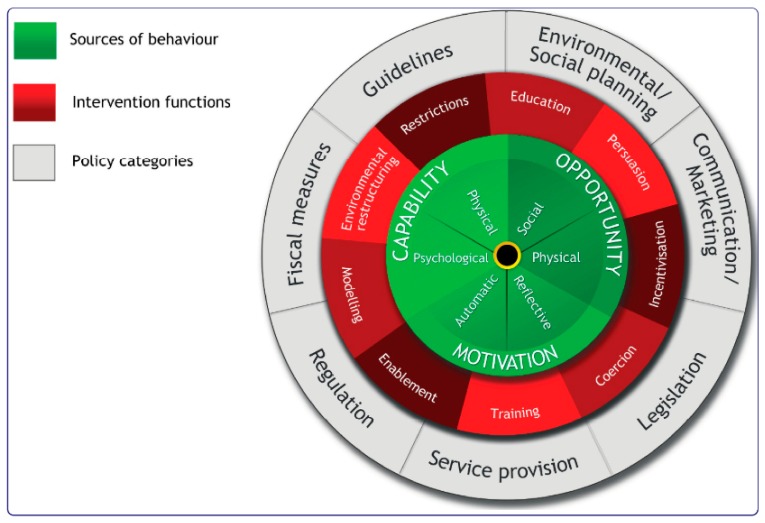
The Behaviour Change Wheel (Michie et al., 2011) [53].

Recent developments in social science have led to systematic guidance and lists of behavioural techniques which provide a mechanism to develop theory-based behaviour change interventions, detail the mechanisms through which change is expected to occur and describe intervention content using shared terminology. Michie et al.’s (2011) [53] Behaviour Change Wheel (see Figure 1) was developed from 19 frameworks of behaviour change, synthesizing the common features of the framework and linking them to a model of behaviour that was sufficiently broad that it could be applied to any behaviour in any setting [59], including animal welfare-promoting behaviours in farm settings. 

The main goal of the Behaviour Change Wheel is to support developers to design theory-based interventions and to increase the replicability and efficacy of behaviour change interventions. According to the COM-B system at the inner-most layer of the wheel, changing any *Behaviour* involves identifying what needs to change in terms of *Capability*, *Opportunity*, and *Motivation* [53]. For example, do pig farmers have the Capability to engage in the necessary physical processes (e.g., skills) or thought processes (e.g., knowledge) to perform a target behaviour, such as identifying a bitten pig? More specifically, do farmers know how and why they should identify and remove bitten pigs from the home pen? Are there sufficient physical Opportunities (e.g., resources) or social Opportunities (e.g., norms) that can support this behaviour? Perhaps farmers’ lack of time is a significant barrier to inspecting individual pigs for early signs of tail damage (physical Opportunity). Could knowledge about normative animal welfare behaviours on other pig farms facilitate regular inspection of pigs? (social Opportunity). Is there sufficient automatic Motivation (e.g., habit) or reflective Motivation (e.g., goals) to facilitate the behavioural target? Financial rewards for farmers who reduce tail-biting prevalence may incentivise the regular inspection of pigs for tail damage to become a habitual practice on-farm (automatic Motivation), and performing behaviours that reduce tail-biting may become a goal for farmers if they reflect on the economic costs of tail-biting (reflective Motivation). By designing the schedule of questions around the Theoretical Domains Framework and understanding the barriers and facilitators in terms of the COM-B model, a comprehensive picture of the target behaviour can be identified. 

### 3.2. Identify Intervention Options 

The next step is to design an appropriate intervention according to the determinants of the target behaviour identified in stage 1 [60]. The second layer of the Behaviour Change Wheel is made up of nine intervention functions, the broad categories of means by which an intervention can change behaviour [53]. The outer layer of the wheel is composed of different policy categories. These are actions that can be taken by responsible authorities, such as a government body or assurance scheme provider, that enable the implementation of the intervention and support behaviour change [53]. Michie et al. (2011) [53] detailed how the components of the COM-B model link to each Intervention Function and how these, in turn, link to the various Policy Categories. For example, an intervention with the function *Education* could aim to increase knowledge and understanding of the causes of tail biting in order to impact farmers’ psychological Capability to engage in the target behaviour (e.g., increasing the nutritional quality of feed). This educational intervention would fall under Communication or Marketing policy categories. In contrast, an intervention with the function of *Coercion* could implement a financial cost for non-compliance with animal welfare legislation, thereby increasing farmers’ reflective Motivation to engage in the target behaviour, through the policy categories of Legislation and Regulation. Once the intervention function and policy category have been decided, the intervention content must be specified and options for implementation are explored. 

### 3.3. Identify Intervention Content and Implementation Options

In the final stage of intervention development, the intervention content and implementation options are selected. Behavioural interventions can be complex and may have more than one active component [54]. For example, consider the following scenario: upon carrying out a number of interviews with pig farmers, the researchers identify low motivation as one of the key barriers to effective tail biting management. A number of behaviour change intervention options are reviewed and it is decided that based on the available evidence, the function of the intervention will be *Incentivisation* in the form of a financial reward to increase farmers’ reflective Motivation to engage in tail biting management behaviour. At the beginning of the study, the farmers in the ‘financial reward’ condition are informed that they will receive a reward if they renew the environmental enrichment material in each pen on a weekly basis (i.e., the target behaviour). The farmers are also informed that a researcher will visit each farm weekly to confirm whether this target behaviour has been performed. Over the following weeks, farmers that have successfully performed the behaviour receive their reward. While this intervention appears to be simply a ‘financial reward’ intervention, there are, in fact, a number of components that may be influencing behaviour. For example, the promise of a reward, the reward itself, or the weekly checks by the researchers could all have influenced the farmer’s behaviour to varying extents. 

The Behaviour Change Technique Taxonomy [54] is a standardized list of 93 behaviour change techniques (BCTs). BCTs are the active ingredients that make up the content of behaviour change interventions [54]. Appropriate BCTs are selected on the basis of their successful application in previous research. The Behaviour Change Technique Taxonomy allows researchers to develop interventions in a systematic and rigorous way. This not only aids the design process but also ensures precise reporting of behaviour change interventions. The intervention developer must specify the precise nature of the reward in terms of the BCT taxonomy. Considering the scenario outlined above, is the reward material or social? Is the behaviour or outcome being rewarded? Is the reward operationalised as a self-reward or a reward given by another person? When the BCT taxonomy is applied to the above scenario, a number of distinct active ingredients are evident in the intervention. Specifically, the BCTs ‘material incentive’ (behaviour), ‘material reward (behaviour)’ and ‘monitoring of behaviour by others without feedback’ [54] are apparent in the example scenario. 

A study carried out by Taylor et al. (2012) [61] highlights the importance of specifying the active ingredients of all aspects of an intervention. This study explored intervention strategies for tail biting on English pig farms. Each farm was visited on at least two occasions between 2007 and 2008 and a tail biting risk assessment was carried out. Farms were allocated to either an ‘Advice’ group, which received tailored tail biting advice during initial and follow-up farm visits, a ‘Financial Incentive’ group that received advice and an incentive to encourage implementation of changes, or a ‘Control’ group in which risk assessments were carried out but were not discussed with the farmers until study completion. Taylor et al. (2012) [61] found that the Financial Incentive group and Control group significantly decreased their tail biting risk scores by the final farm visit. However, there was no difference in the risk scores from the first to the final visit for the Advice treatment group. This surprising finding may be due in part to the fact that the ‘Control’ group was, in fact, also receiving a type of intervention; the Hawthorne Effect is a phenomenon by which individuals often change their behaviour when they know that they are being observed [62,63]. When the content of the study conditions in Taylor et al. (2012) [61] are mapped to the Behaviour Change Technique Taxonomy [54], we may predict that participants in the Control group are receiving BCTs such as, ‘monitoring of behaviour by others without feedback’ [54]. It is possible that the BCTs delivered in the Advice condition were less effective for reducing tail-biting risk than those delivered in the Financial Incentive and Control conditions. However, as the researchers did not use the BCT taxonomy to specify the content of treatment or control conditions, this interpretation of the results remains speculative. 

This mapping of intervention content to the BCT taxonomy makes interventions easier to replicate in future studies and makes it easier to compare interventions within systematic reviews [54]. The BCT Taxomomy v1 is available to download as a free smartphone app [64]. The smartphone app provides guidance notes under each intervention category to ensure that all active components of the intervention are correctly documented. This guidance is also available to download as supplementary material from Michie et al. (2013) [54]. This guidance increases the accessibility of the Behaviour Change Technique Taxonomy to researchers outside of the social sciences, or those unacquainted with theories of behaviour change. Table 2 provides an example of how behaviour change interventions for reducing tail biting can be developed by applying principles from the Theoretical Domains Framework [52], the Behaviour Change Wheel [53] and the Behaviour Change Techniques Taxonomy [54].

## 4. Application of Behavioural Science Theory in Animal Welfare and Agricultural Research to Date

To date, the Behaviour Change Wheel and associated theories have been utilised to varying extents in a number of animal welfare and farmer behaviour change studies. For example, Mohite et al. (2019) [65] examined barriers to technical competence in Indian farriers via focus groups. Mohite et al. (2019) [65] concluded that due to unwillingness of horse owners to pay for a good service and a low motivation in the farriers for training, behaviour change interventions should target Opportunity and Motivation. However, the focus group discussions did not appear to have been shaped around the COM-B model and the model was referred to only once, in the discussion section of the paper. Similarly, McLeod et al. (2015) [66], in their study on barriers to and drivers of cat containment, referred to Michie et al. [53,54] in their discussion of possible intervention strategies to reduce the population of free-roaming cats in Tasmania, Australia. However, pet-owner questionnaires were based on a mixture of cat and dog management literature, behaviour theory and social psychology rather than following any one psychological framework. McLeod et al. (2015) [66] also acknowledge that a number of drivers and barriers identified by Michie et al. (2013) [54] were not explored. 

To date, a small number of studies related to animal welfare have used the Behaviour Change Wheel to develop intervention strategies. For example, Malik and Bradbury (2016) [67] used the COM-B model, the inner-most layer of the Behaviour Change Wheel, to develop a number of potential methods that veterinary nurses can use to increase analgesia use in small veterinary practices. Barriers to a behaviour, specifically adequate analgesia use, were identified within the literature and mapped to Capability, Opportunity and Motivation. For instance, Malik and Bradbury (2016) [67] stated that Capability may be reduced by an inability to identify signs of pain, while opportunity may be influenced by owner budget considerations. In addition, motivation to administer analgesia may be influenced by the perceived moral status of the animal being treated; a pet may receive more analgesia than a pest or wild animal species. Malik and Bradbury (2016) [67] made a number of recommendations based on this mapping exercise, such as increasing pain assessment and counteracting species prejudice. However, the effectiveness of the suggested interventions was not assessed.

In order to address a lack of flock health services by veterinarians, Bellet at al. (2015) [68] explored veterinarian’s beliefs surrounding their current provision of disease prevention advice for sheep farmers. Interviews and questionnaires were used to identify common themes that explain these beliefs. In the discussion section, these themes were mapped to the COM-B model. However, the model was not used in the study design phase and was first referred to in the discussion section of the paper. In addition, the method used to design the interview and questionnaire questions was unclear. The Theoretical Domains Framework [52] may have been useful in this situation as questions could have been designed around domains known to influence behavior, such as knowledge, skills and professional role, and identity [52]. In any case, it is encouraging that there is an increased awareness among scientists, outside the discipline of health psychology, of the existence of overarching frameworks of behaviour change; King et al. (2018) [69] carried out explorative interviews with veterinary surgeons on appropriate antibiotic prescribing behaviour and reported that they intended to use the information gathered to design behaviour change interventions using the Theoretical Domains Framework, the Behaviour Change Wheel and the Behaviour Change Technique Taxonomy in future studies [52,53,54]. Furthermore, authors exploring human behaviour change with regard to equine welfare and farm biosecurity [70,71,72] acknowledge the benefits of using multifaceted approaches to human behaviour change that consider both internal and external influences on human behaviour. In one of the more comprehensive animal welfare studies that use the Behaviour Change Wheel, McDonald et al. (2018) [73] sought to increase reporting of stray cats for neutering in an English town with an established problem with stray cats. Barriers to and enablers of reporting stray cats were identified by conducting a series of face-to-face surveys, which were then mapped to the Behaviour Change Wheel. Based on these barriers and enablers, community members’ Opportunity and Capability to increase reporting were targeted by providing a variety of ways of reporting stray cats and by creating a presence within the community via community hub drop-in sessions and events, respectively. Motivation (reflective) was targeted by communicating the benefits of having a stable neutered cat population [73,74]. The trap-neuter-release was successful in increasing the number of stray cats that were reported. However, there was no control group used in this study for comparison. There is a need for more animal welfare research that incorporates a randomized control trial design. Furthermore, animal welfare research may be enhanced by adopting a more systematic, rigorous, and theory-based approach to intervention development. It is common practice in health behaviour change research to conduct pilot studies prior to a full-scale randomized control trial evaluating effectiveness. For example, the Cardiac Health and Relationship Management and Sexuality (CHARMS) intervention study described a sexual education and support intervention for cardiac patients designed using the Behaviour Change Wheel [75]. This intervention was found to be feasible in practice [76] and acceptable to recipients [77]. There is also evidence that the Behaviour Change Wheel and related theories can be employed to create effective behaviour change interventions. For example, The Football Fans in Training programme was designed using the principles of behavioural science [78]. In the development phase, the intervention content was mapped to the BCT taxonomy. This programme, delivered through professional football clubs using Behaviour Change Techniques, was found to be effective for weight loss [79].

It should be noted that while these frameworks provide a guide for designing behaviour change interventions, the success of any intervention depends on the specialist knowledge of the developer. For example, with regard to the target behaviour (such as removal of the biter when tail biting occurs), Atkins et al. (2017) [55] described a number of considerations: 1. how modifiable the behaviour is likely to be (how easy is it to identify the biter? Will farmers have time to observe pigs to ascertain this? Is this behaviour sustainable?), 2. how important the behaviour is in bringing about change (how successful is this strategy for reducing tail biting compared to other methods?), 3. how change in the target behaviour may affect related behaviour (will time spent identifying the biter take time away from other important activities such as health monitoring?), and 4. how easy the target behaviour is to measure (how can you determine whether the farmer is actually identifying and removing the biter from the pens?). If this is not given adequate consideration, regardless of the theoretical framework applied, the intervention is unlikely to succeed. However, the advantage of using the Behaviour Change Wheel is the simplicity and clarity of the COM-B system. This is especially relevant in multi-disciplinary teams where there may be a range of theories or frameworks to choose from. The COM-B system provides a simple framework suitable for application to any behaviour in any setting. It is hoped that this guide has provided a solid starting point for the development of behaviour change interventions in the field of Animal Welfare Science and will encourage further research in this area. 

## 5. Conclusions

Tail biting has been deemed one of the most harmful behaviours pigs perform. Despite decades of research, there has been little change in tail biting prevalence within intensive pig herds. While tail biting will always exist in commercial farming systems to an extent, understanding the reasons why farmers act or fail to act to improve animal welfare is key if tail biting is to be successfully tackled. Interventions targeting farmer behaviour must be developed and adopting a theory-based approach to intervention design is critical. Behaviour change interventions informed by theory are more effective than those that are not. Interventions based on the Theory of Planned Behaviour have particularly strong effects on behaviour. However, the theory has been criticised, identifying major limitations of the theory in practice [39,40]. The Behaviour Change Wheel is different from traditional theories, such as the Theory of Planned Behaviour, because it considers the role of context, an aspect of behavior, which has been under-investigated in the past. Within this system, internal and external forces that influence behaviour are given equal consideration.

The Theoretical Domains Framework can be used to identify barriers to and facilitators of behaviour change by informing the schedule of questions included in questionnaires, focus groups and interviews. The Behaviour Change Wheel then provides a useful way of linking a model of behaviour to common functions of interventions to change that behaviour (e.g., education, persuasion, coercion, incentivization), and in turn, linking these intervention functions to policy categories (e.g., legislation, communication) that facilitate behaviour change. The Behaviour Change Technique Taxonomy can then be used to specify intervention content. The Theoretical Domains Framework, Behaviour Change Wheel and Behaviour Change Technique Taxonomy support intervention developers in designing theory-based behaviour change interventions. Greater use of the theory-based elements and standardised frameworks, such as those described above, makes interventions easier to replicate in future studies and allows comparisons to be made between studies. Despite evidence that using BCTs increases the efficacy of behaviour change interventions, the Behaviour Change Wheel has not been readily applied to interventions to change farmers’ behaviour with the goal of improving animal welfare. This is likely due to a lack of awareness in animal welfare scientists of recent developments in the field of human behaviour change. It is hoped that the guide provided here will prompt greater use of theory-based interventions to reduce tail biting prevalence—a task that is important now more than ever given the likeliness of greater enforcement of the tail docking ban in the near future. 

## Figures and Tables

**Table 1:** A summary of the main features and uses of the Theoretical Domains Framework (TDF) [52], Behaviour Change Wheel (BCW) [53] and the Behaviour Change Techniques Taxonomy (BCTT) [54].

Framework	Main Features	Useful for
Theoretical Domains Framework	128 theoretical constructs from 33 theories of behaviour and behaviour change clustered into 14 domains The 14 theoretical domains are; knowledge; skills; social/professional role and identity; beliefs about capabilities; optimism, beliefs about consequences; reinforcement; intention; goals; memory, attention and decision processes; environmental context and resources; social influences; emotion and behavioural regulation	Systematic identification of potential barriers to and facilitators of a desired behaviour
Behaviour Change Wheel	Three layers comprised of; COM-B model: Provides the basis for understanding behaviour in context and selecting appropriate intervention function Intervention functions: Broad categories of means by which an intervention can change behaviour Policy categories: Actions that can be taken by responsible authorities that enable implementation	Understanding a behaviour Designing theory-based interventions to change behaviour Increasing the replicability and efficacy of behaviour change interventions
Behaviour Change Techniques Taxonomy	A comprehensive standardized list of 93 behaviour change techniques (BCTs) grouped into 16 clusters The 16 clusters are; goals and planning; feedback and monitoring; social support; shaping knowledge; natural consequences; comparison of behaviour; associations; repetition and substitution; comparison of outcomes; reward and threat; regulation; antecedents; identity; scheduled consequences; self-belief; covert learning	Creating intervention content Specifying the active ingredients of behaviour change interventions Increasing the replicability and efficacy of behaviour change interventions

**Table 2:** Applying principles from the Behaviour Change Wheel [53], the Theoretical Domains Framework [52] and the Behaviour Change Techniques Taxonomy [54] to design a behavioural intervention for farmers to reduce tail biting among their pigs.

Barrier to Behaviour	Theoretical Domain	COM-B (Source of Behaviour)	Intervention Function	Policy Category	Behaviour Change Technique	Intervention Description
Lack of knowledge about how to prevent tail biting	Knowledge	Capability (psychological and physical) Motivation (reflective)	Education	Guidelines	Instruction on how to perform a behaviour	A document outlining recommended practices to reduce tail biting are disseminated to farmers
Not concerned with animal welfare	Reinforcement	Motivation (automatic)	Incentivisation	Fiscal measures	Material reward (behaviour)	Inform the farmer that they will receive money if behavioural target (e.g., adding environmental enrichment) has been performed
Unaware that tail biting has negative economic consequences	Knowledge Belief about consequences	Motivation (reflective) Capability (psychological)	Education Persuasion	Communication/Marketing	Information about social and environmental consequences Credible source	A video is shown detailing relationship between tail lesions and economic loss. A well-respected celebrity farmer presents the information
Financial Constraints	Environmental Context and Resources	Capability (physical) Opportunity (physical)	Enablement	Fiscal measures	Material incentive (behaviour)	Farmers are given financial support to implement behavioural targets (e.g., vouchers to purchase enrichment material)
Time constraints	Environmental context and resources Skills	Opportunity (physical) Capability (physical)	Modelling Training	Service provision	Demonstration of the behaviour Social support (practical)	Experienced peer demonstrates how to incorporate tail biting management into daily routine

Behavioural targets = 1. Environment enrichment; 2. Removing bitten pig.
